# Comment on Orozco-Beltrán et al. Evaluation, Management and Therapeutic Approach of Cardiovascular–Kidney–Metabolic Syndrome: A Multidisciplinary Delphi Expert Consensus. *J. Clin. Med.* 2025, *14*, 8930

**DOI:** 10.3390/jcm15176637

**Published:** 2026-08-28

**Authors:** Javier Crespo, Paula Iruzubieta

**Affiliations:** 1 Valdecilla Research Institute (IDIVAL), 39008 Santander, Spain; 2 School of Medicine, University of Cantabria, 39008 Santander, Spain; 3 MedicineAI, 28028 Madrid, Spain; 4 Gastroenterology and Hepatology Department, Marqués de Valdecilla University Hospital, 39008 Santander, Spain; paula.iruzubieta@scsalud.es; 5 CIBER of Hepatic and Digestive Diseases (CIBERehd), Instituto de Salud Carlos III, 28028 Madrid, Spain

The multidisciplinary consensus by Orozco-Beltrán and colleagues [1] is a rigorous and necessary contribution to the integrated management of the cardiovascular–kidney–metabolic (CKM) syndrome within the Spanish National Health System. Since its publication, several developments have reinforced the relevance of liver health within integrated cardiometabolic medicine: on 21 May 2026, the Seventy-Ninth World Health Assembly adopted the resolution “Steatotic liver disease: a missing piece in the global noncommunicable disease response”—the first formal recognition of a liver disease within the global noncommunicable disease (NCD) agenda—with a mandate for Member States, Spain among them, to integrate liver health into their national strategies by the end of 2026 [2]. Our aim is not to judge the consensus in hindsight against this subsequent evidence but to build on it: its hepatic dimension, we suggest, offers a timely opportunity for refinement, and the reason it has not yet been fully developed is, in part, structural.

Before turning to that dimension, the strengths of the consensus warrant emphasis. Its multidisciplinary Delphi design, the breadth of participation across five specialties, the high response rate among panelists, and its deliberate orientation toward practical implementation within a single national health system make it an unusually actionable document. The observations that follow are offered in that spirit: as an extension that builds on a solid foundation, not as a rebuttal.

The Delphi panel comprised seventy experts from five specialties—thirteen cardiologists, fourteen nephrologists, twelve endocrinologists, twelve internists, and nineteen primary care physicians [1]—a composition that closely mirrors the organ systems named in the original American Heart Association CKM construct [3], which did not explicitly incorporate the liver. The choice is, in that sense, understandable: the panel reflected the conceptual boundaries of the framework it set out to operationalize at a moment when the CKM field was still evolving. One consequence may be that the liver is incorporated primarily through laboratory assessment rather than through a fully operational, organ-based pathway.

This pattern is visible in three precise places. The Fibrosis-4 index (FIB-4) enters only in the basic laboratory panel (statement 7) and is not taken up again in the operational architecture. Hepatic steatosis does not figure among the criteria defining the metabolic component of the syndrome (statement 12). And MASLD, while explicitly acknowledged in the Discussion as a “future action area”—a recognition we welcome—is not yet carried into the body of diagnostic, staging, and therapeutic statements that constitute the consensus.

This gap sits in tension with the conceptual architecture of the field itself. In 2023, a multisociety Delphi panel—comprising 236 experts from 56 countries, convened under the three major hepatology associations—defined the disease by anchoring it in the presence of at least one of five cardiometabolic risk factors, chosen precisely because they were already validated in the context of cardiovascular disease [4]. The definition of MASLD was thus built on the very same cardiometabolic substrate that structures the CKM syndrome. Treating hepatic steatosis as external to the metabolic component of that syndrome, when contemporary hepatology defines it on exactly those criteria, therefore creates a tension with the prevailing taxonomy. The point sharpens when one recalls that the consensus itself places adipose tissue dysfunction as an initial driver of the syndrome (statement 3): the steatotic liver is a paradigmatic destination of that ectopic fat and an active endocrine organ whose dysregulated hepatokine secretion feeds back on insulin resistance, systemic inflammation, and vascular dysfunction.

The handling of FIB-4 illustrates the opportunity most clearly. Placing a fibrosis index in first-line screening already acknowledges that advanced fibrosis is a prognostic signal worth detecting early; the proposal here is simply to carry that logic through to its operational conclusion. As it stands, the consensus does not specify interpretive thresholds (1.3 and 2.67, with an age-adjusted lower cut-off in those over 65 years), a second-line test, or a referral pathway, nor does it link the result to the stages of the syndrome or to therapeutic targets. The prognostic signal at stake is of the first order: in the prospective study of the NASH Clinical Research Network published in the *New England Journal of Medicine*, all-cause mortality rose stepwise with fibrosis stage—approximately 0.32, 0.89, and 1.76 deaths per 100 person-years across early fibrosis (F0–F2), bridging fibrosis (F3), and cirrhosis (F4) [5]—and a separate long-term histologic cohort confirmed that fibrosis stage, rather than steatosis or inflammation, is the feature that governs outcome [6]. Cardiovascular disease, moreover, remains the leading cause of death across the cardiometabolic spectrum [3], and a liver fibrosis marker independently predicts cardiovascular morbidity and mortality in the general population, even after adjustment for conventional risk scores [7]. The liver thus behaves as a sentinel of cumulative cardiometabolic burden [8]; an actionable FIB-4 pathway would convert that signal into care rather than leave it unread.

Two clarifications frame the proposals that follow. First, and importantly, we do not propose modification of the CKM terminology itself; rather, we advocate a more robust integration of the hepatic axis within the existing CKM framework. The broader, longer-term debate over expanding the construct toward a cardiovascular–renal–hepatic–metabolic continuum is legitimate but separate [9], and our proposal does not depend on it. Second, we distinguish between what could reasonably be amended in the present consensus and what might be reserved for future iterations of CKM guidance, as summarized in Table 1.

These observations are not a disciplinary reproach; they describe how a panel’s composition shapes its output, and they point toward amendments that can be made with instruments that already exist. We propose three. First, hepatic steatosis in the context of metabolic dysfunction should be recognized as a constitutive criterion of the metabolic component of the syndrome rather than as an external comorbidity, consistent with the current international definition of the disease [4]. Second, FIB-4 should be operationalized into a stepwise, risk-proportionate pathway. It need not be designed from scratch: the EASL-EASD-EASO clinical practice guideline already specifies it—FIB-4 at the first level and transient elastography or the ELF test at the second, with specialist referral reserved for a high probability of advanced fibrosis, pharmacotherapy confined to significant fibrosis (≥F2), and lifestyle management in its absence [10]—and it can be embedded in the same electronic health record alert architecture the consensus already advocates for estimated glomerular filtration rate and albuminuria. Third, advanced fibrosis should be treated as a systemic risk marker of the same rank as microalbuminuria, glycated hemoglobin, or LDL cholesterol and incorporated accordingly into staging, therapeutic targets, and quality indicators.

Operationalizing FIB-4 within Spanish primary care raises practical considerations that a pathway should address explicitly rather than leave implicit. FIB-4 loses specificity with advancing age and generates false positives in older adults; the EASL-EASD-EASO guideline accordingly raises the lower threshold to 2.0 beyond the age of 65 [10]. The two-step design is itself a workload-containment strategy: only the minority with an indeterminate or elevated FIB-4 proceed to second-line testing, and only those with a high probability of advanced fibrosis are referred, which protects both elastography capacity and hepatology clinics. Where point-of-care transient elastography is unavailable, the ELF test or centralized “one-stop” elastography circuits offer feasible alternatives [10,11]. Finally, because the consensus already advocates electronic health record alerts for estimated glomerular filtration rate and albuminuria, the same infrastructure can host the FIB-4 pathway at marginal cost, embedding interpretation and referral logic at the point of care. These are the conditions under which the pathway becomes realistic within the Spanish National Health System.

These proposals do not arise in a vacuum. The second EASL-Lancet Commission has charted a framework for the population-level implementation of liver health in Europe [11]; the World Health Assembly resolution has given it the rank of a policy mandate [2]; an expansion of the CKM construct toward a cardiovascular–renal–hepatic–metabolic continuum has been proposed [9]; and that program has begun to be articulated for the Spanish context [12,13,14]. This convergence places the CKM consensus, in its hepatic dimension, in a position that is as straightforward to strengthen as it is timely. None of this diminishes the value of the work by Orozco-Beltrán and colleagues, which is rigorous and necessary; our aim is to complement and extend it. The liver, the heart, and the kidney are coordinated expressions of a single metabolic state, and a framework conceived to govern that state in an integrated manner is most complete when the hepatic axis is afforded operational parity with the renal and cardiovascular axes. To recognize, measure, and act upon liver fibrosis is, in this sense, not an appendix to the care of the CKM syndrome but a natural extension of it.

## Figures and Tables

**Table 1:** Proposed enhancements to the current CKM consensus framework.

Current Limitation in the Consensus	Clinical Consequence	Proposed Amendment
Hepatic steatosis is absent from the criteria defining the metabolic component (statement 12); MASLD is confined to the Discussion as a future action area.	A principal destination of ectopic fat is treated as an external comorbidity, fragmenting metabolic risk assessment.	Recognize metabolic dysfunction–associated hepatic steatosis as a constitutive criterion of the metabolic component, consistent with the international MASLD definition [4].
FIB-4 appears only within the basic laboratory panel (statement 7), without thresholds, a second-line test, or a referral pathway.	A first-order prognostic signal is captured and then left unacted upon; advanced fibrosis goes undetected.	Operationalize FIB-4 as a stepwise, age-adjusted pathway (FIB-4 → transient elastography/ELF → specialist referral), embedded in EHR alerts, per the EASL-EASD-EASO guideline [10].
Advanced fibrosis is not incorporated into staging, therapeutic targets, or quality indicators.	The hepatic axis is granted a lesser operational status than the renal and cardiovascular axes despite comparable prognostic weight.	Treat advanced fibrosis as a systemic risk marker of the same rank as microalbuminuria, HbA1c, or LDL cholesterol, and incorporate it into staging, targets, and quality indicators.

## Data Availability

No new data were created or analyzed in this study. Data sharing is not applicable to this article.
